# Association of *CDH13* Genotypes/Haplotypes with Circulating Adiponectin Levels, Metabolic Syndrome, and Related Metabolic Phenotypes: The Role of the Suppression Effect

**DOI:** 10.1371/journal.pone.0122664

**Published:** 2015-04-13

**Authors:** Ming-Sheng Teng, Lung-An Hsu, Semon Wu, Yu-Chen Sun, Shu-Hui Juan, Yu-Lin Ko

**Affiliations:** 1 Graduate Institute of Medical Sciences, Department of Physiology, College of Medicine, Taipei Medical University, Taipei, Taiwan; 2 Department of Research, Taipei Tzu Chi Hospital, Buddhist Tzu Chi Medical Foundation, Taipei, Taiwan; 3 The First Cardiovascular Division, Department of Internal Medicine, Chang Gung Memorial Hospital and Chang Gung University College of Medicine, Taoyuan, Taiwan; 4 Department of Life Science, Chinese Culture University, Taipei, Taiwan; 5 Department of Laboratory Medicine, Chang Gung Memorial Hospital, Taoyuan, Taiwan; 6 The Division of Cardiology, Department of Internal Medicine and Cardiovascular Medical Center, Taipei Tzu Chi Hospital, Buddhist Tzu Chi Medical Foundation, Taipei, Taiwan; 7 School of Medicine, Tzu Chi University, Hualien, Taiwan; Johns Hopkins Bloomberg School of Public Health, UNITED STATES

## Abstract

**Objective:**

Previous genome-wide association studies have indicated an association between *CDH13* genotypes and adiponectin levels. In this study, we used mediation analysis to assess the statistical association between *CDH13* locus variants and adiponectin levels, metabolic syndrome, and related metabolic phenotypes.

**Methods and results:**

A sample population of 530 Taiwanese participants was enrolled. Four *CDH13* gene variants in the promoter and intron 1 regions were genotyped. After adjustment for clinical covariates, the *CDH13* genotypes/haplotypes exhibited an association with the adiponectin levels (lowest *P* = 1.95 × 10^−11^ for rs4783244 and lowest *P* = 3.78 × 10^−13^ for haplotype *ATTT*). Significant correlations were observed between the adiponectin levels and the various metabolic syndrome-related phenotypes (all *P* ≤ 0.005). After further adjustment for the adiponectin levels, participants with a minor allele of rs12051272 revealed a considerable association with a more favorable metabolic profile, including higher insulin sensitivity, high-density lipoprotein cholesterol levels, lower diastolic blood pressure, circulating levels of fasting plasma glucose, and triglycerides, and as a lower risk of metabolic syndrome (all *P* < 0.05). The mediation analysis further revealed a suppression effect of the adiponectin levels on the association between *CDH13* genotypes and metabolic syndrome and its related phenotypes (Sobel test; all *P <* 0.001).

**Conclusion:**

The genetic polymorphisms at the *CDH13* locus independently affect the adiponectin levels, whereas the adiponectin levels exhibit a suppressive effect on the association between *CDH13* locus variants and various metabolic phenotypes and metabolic syndrome. In addition, these results provide further evidence of the association between the *CDH13* gene variants and the risks of metabolic syndrome and atherosclerotic cardiovascular disease.

## Introduction

Adiponectin is one of the most abundant gene products expressed in adipose tissues and plays a crucial role in the metabolic regulation of obesity, insulin sensitivity, and atherosclerosis [1]. Several studies have indicated many metabolic actions of adiponectin, such as antidiabetic, antiinflammatory, and antiatherosclerotic actions [2]. Animal studies and cell culture experiments revealed that a direct stimulation of nitric oxide synthesis is responsible for the antiinflammatory and antiatherogenic effects of adiponectin [3]. Decreased levels of plasma adiponectin have been associated with an increased risk of obesity, metabolic syndrome, and atherosclerotic cardiovascular disease [4–7]. Genetic factors have also been suggested to regulate adiponectin levels, as demonstrated by the twin study [8], family study [9], and genome-wide linkage scans [10], which indicated moderate to high estimates of heritability (30%–70%) [11]. In addition, a recent family-based study reported a shared heritability between adiponectin levels and metabolic syndrome [12].

Various genome-wide association studies conducting meta-analysis have reported numerous candidate gene loci for adiponectin levels [13–16]. T-cadherin, belonging to the cadherin superfamily of the transmembrane proteins that mediate calcium-dependent intercellular adhesion, is the receptor for hexameric and high-molecular-weight (HMW) adiponectin expressed in the vasculature [17] and cardiac myocytes [18]. The *CDH13* gene, encoding T-cadherin, is localized at chromosome 16q23.3, spans 1.2 Mb, and contains 14 exons. A meta-analysis revealed the *CDH13* gene region to be the most crucial locus associated with adiponectin levels [16]. By contrast, other studies have reported a marked association between *CDH13* gene variants and various metabolic phenotypes but with controversial results [14, 15, 19]. In a Taiwanese study, the adiponectin-lowering T allele was paradoxically associated with a reduced risk of diabetes, metabolic syndrome, and stroke [14]. Two recent reports on East Asian populations have revealed that *CDH13* intron 1 polymorphisms were associated with metabolic phenotypes only after an adjustment for adiponectin levels [15, 19]. In the present study, we used mediation analysis to further elucidate the relationships of *CDH13* gene variants with circulating adiponectin levels, metabolic phenotypes, and metabolic syndrome.

## Participants and Methods

### Study population

This study was approved by the institutional review board of Taipei Tzu Chi Hospital, Buddhist Tzu Chi Medical Foundation (IRB number: 02-XD56-120). A total of 617 Han Chinese subjects (327 men, 290 women) responded to a questionnaire on their medical history and lifestyle characteristics were recruited during routine health examinations between October 2003 and September 2005 at Chang Gung Memorial Hospital. All of the participants provided written informed consent. The exclusion criteria included cancer, current renal or liver disease, and a history of myocardial infarction, stroke, or transient ischemic attacks. After initial recruitment, 87 individuals were further excluded from the analysis in this investigation, with either participants aged < 18 years (5 subjects), or with a history of regular use of medications for diabetes mellitus, hypertension, and/or lipid-lowering drugs (82 subjects). In total, 530 study participants were enrolled for analysis (mean ± standard deviation [SD]): 270 men, age = 43.9 ± 9.3 years; 260 women, age = 45.9 ± 9.3 years. Table 1 summarizes the clinical and biometric features of the study group. The participants responded to a questionnaire on their medical history and lifestyle characteristics and underwent a physical examination that involved measurement of height, weight, waist and hip circumference, and blood pressure (BP) in the sitting position after a 15-min rest period. Fasting blood samples were obtained from each participant. Metabolic syndrome characteristics were based on the recent update of the third report of the National Cholesterol Education Program's Adult Treatment Panel III (ATP-III) criteria [20]. According to the ATP-III criteria, subjects with 3 or more of the following attributes are typically defined as having MetS: (1) BP of at least 130/85 mm Hg and/or taking medication for hypertension; (2) triglycerides of at least 150 mg/dL; (3) HDL-C less than 40 mg/dL for men and less than 50 mg/dL for women; (4) fasting plasma glucose of at least 100 mg/dL and/or taking medication for diabetes mellitus; and, (5) waist circumference greater than 90 cm for men and greater than 80 cm for women (modified criteria for Asians) [21]. Hypertension was defined as a systolic BP≥140 mm Hg, a diastolic BP≥90 mm Hg, or both, or the regular usage of antihypertensive drugs. Diabetes mellitus was defined as fasting plasma glucose levels before a meal of ≥7.0 mmol/l or the regular use of medication for the treatment of diabetes mellitus. Obesity was defined as a body mass index (BMI) ≥ 25 kg/m2, according to the Asian criteria [21]. Current smoker was defined as smoking at least 1 cigarette per day at the time of survey.

**Table 1:** Baseline characteristics of the health examination participants.

	Total	Men	Women	P values
Number	530	270	260	
Age (years)	44.87 ± 9.38	43.88 ± 9.32	45.90 ± 9.34	0.013
Systolic BP (mm Hg)	112.82 ± 16.07	113.66 ± 14.25	111.96 ± 17.76	0.223
Diastolic BP (mm Hg)	75.05 ± 10.08	76.95 ± 9.81	73.08 ± 10.0	<0.001
Total cholesterol (mg/dL)	199.25 ± 36.28	202.16 ± 36.24	196.23 ± 36.14	0.060
HDL cholesterol (mg/dL)	55.82 ± 14.47	49.90 ± 12.07	61.97 ± 14.20	<0.001
LDL cholesterol (mg/dL)	116.62 ± 33.17	120.13 ± 34.28	112.97 ± 31.63	0.013
Triglycerides (mg/dL)	138.54 ± 110.61	168.68 ± 136.56	107.2 ± 60.74	<0.001
Body mass index (kg/m^2^)	24.15 ± 3.49	24.78 ± 3.17	23.50 ± 3.69	<0.001
Fasting plasma glucose (mg/dL)	95.35 ± 22.22	97.80 ± 25.38	92.80 ± 18.06	0.009
Fasting serum insulin (μU/mL)	9.06 ± 4.93	9.57 ± 5.64	8.53 ± 4.00	0.015
HOMA-IR index	2.16 ± 1.36	2.33 ± 1.58	1.98 ± 1.07	0.003
QUICKI	0.349 ± 0.025	0.345 ± 0.025	0.352 ± 0.024	0.002
Adiponectin (mg/L)	7.25 ± 4.90	5.45 ± 3.44	9.13 ± 5.45	<0.001
Waist circumference (cm)	84.57 ± 9.66	87.41 ± 7.76	81.61 ± 10.53	<0.001
Diabetes mellitus (%)	2.5	2.6	2.3	0.832
Current smokers (%)	19.6	34.8	3.8	<0.001
Hypertension (%)	9.6	8.9	10.4	0.559
Obesity (%)	36.6	44.1	28.8	<0.001
Metabolic syndrome (%)	14.3	15.2	13.5	0.571
Insulin resistance (%)	22.5	24.4	20.4	0.263

BP, blood pressure; HDL, high-density lipoprotein; LDL, low-density lipoprotein; HOMA-IR, homeostasis model assessment of insulin resistance; QUICKI, quantitative insulin sensitivity check index; Continuous variables are presented as mean ± standard deviation (SD).

### Genomic DNA extraction and genotyping

Genomic DNA was extracted as reported previously [22]. Four single-nucleotide polymorphisms (SNPs) in the promoter and intron 1 regions of the *CDH13* gene, which were previously reported to exhibit strong associations with adiponectin levels or metabolic syndrome, were examined in this study (Table A in S1 File). Genotyping were performed using TaqMan SNP Genotyping Assays obtained from Applied Biosystems (ABI, Foster City, CA, USA).

### Laboratory examinations and assays

The laboratory examinations and assays were performed in accordance with the methods described by Hsu et al. [22]. The homeostasis model assessment of insulin resistance (HOMA-IR) index, used as the measurement for insulin resistance, was calculated by using the following formula: fasting serum insulin (μU/ml) × fasting plasma glucose (mmol/l) /22.5 [23]. Insulin resistance was recognized when the HOMA index reached the upper quartile. Quantitative insulin sensitivity check index (QUICKI), used as the measurement for insulin sensitivity, is defined as follows: QUICKI = 1/[(log(I0)+log(G0)], where I0 is the fasting plasma insulin level (μU/ml) and G0 is the fasting blood glucose level (mg/dl) [24]. Serum adiponectin levels were measured by an in-house sandwich enzyme immunoassay. Our kits compared well with the commercial kits for adiponectin (R&D Systems, Minneapolis, MN, USA), with correlation coefficients of 0.98. The within-day precision and day-to-day precision were 7.7% and 7.0% for adiponectin.

### Statistical analysis

Statistical analysis was performed as previously described [25]. The analysis of deviation from the Hardy–Weinberg equilibrium, an estimation of the linkage disequilibrium between polymorphisms, was performed using the Golden Helix SVS Win32 7.3.1 software. The values of the high-density lipoprotein (HDL) cholesterol, low-density lipoprotein (LDL) cholesterol, total cholesterol, triglyceride, and adiponectin levels were logarithmically transformed prior to statistical analysis to adhere to a normality assumption. In addition, stepwise linear regression analysis was used to determine the independent predictors of the adiponectin levels with age, sex, body mass index, and current smoking status as confounding covariates. To explore the mediation effects of the adiponectin levels on the association between *CDH13* gene variants and the metabolic phenotypes, a conceptual model was hypothesized for the test, and four criteria were suggested to evaluate the suppression effects of adiponectin levels [25]. **Criterion** one, independent variable (*CDH13* genotype) must predict the mediator (adiponectin level). **Criterion** two, the mediator (adiponectin level) must predict the dependent variable when adjusting for independent variable. The mediation effect was calculated as the product of the two regression coefficients from **criterion** one and **criterion** two, and reflected the intermediate pathways from independent variable to mediator and in turn to dependent variable. The regression coefficient relating independent variable to dependent variable adjusting for the mediator was expressed as direct effect. **Criterion** three, independent variable must have a significant effect on dependent variable, which was expressed as total effect. The total effect can also be obtained by summation of direct and mediation (indirect) effects. **Criterion** four, the mediation effect must be significant using the procedure outlined by Sobel [26, 27]. In addition, a suppression effect may be indicated in a situation when the direct effect is larger than the total effect [28]. In this situation, the direct and indirect effects often have fairly similar magnitudes and opposite signs, which may entirely or partially cancel each other out and result in zero or a nonzero but insignificant total effect [29]. Sobel test [30] is the most commonly employed method for examining the statistical significance of mediation effect. We further used the β coefficients and standard errors from the model above to conduct a Sobel test for mediation. The Sobel test was performed using a tool for mediation tests (http://www.quantpsy.org/sobel/sobel.htm), in which the null hypothesis H_0_: **αβ** = 0 is tested. The test statistic S, which is approximately distributed as Z (Eq 2), is obtained by dividing the estimated mediation effect (**αβ)** by the standard error (**δ)** in Eq 1. The reported *p*-values are drawn from the unit normal distribution under the assumption of a Z value of the hypothesis that the mediated effect equals to zero in the population. +/- 1.96 are the critical values of the test ratio which contain the central 95% of the unit normal distribution.

$$\delta^{2}{}_{\alpha \beta }=\delta^{2}{}_{\alpha }\beta^{2}+\delta^{2}{}_{\beta }\alpha^{2}$$ Equation 1

$$Z=\alpha \beta /SQRT(\delta^{2}{}_{\alpha }\beta^{2}+\delta^{2}{}_{\beta }\alpha^{2})$$ Equation 2

**SQRT:** square root

## Results

### Clinical and biochemical characteristics

Table 1 summarizes the demographic features, clinical profiles, and levels of biomarkers for all of the studied health examination participants. No considerable deviation from the Hardy–Weinberg equilibrium was detected for the studied polymorphisms (Table A in S1 File). All of the four studied polymorphisms were in strong pairwise linkage disequilibrium (Table B in S1 File).

### Associations between CDH13 genotypes/haplotypes and circulating adiponectin levels

After adjustment for clinical covariates, significant associations were observed between the adiponectin levels and the studied polymorphisms (Table 2).

**Table 2:** *CDH13* genotypes and adiponectin levels.

*CDH13* genotypes		Adiponectin levels Means ± SD (N)	P value	P*°*value
rs11646213	AA	6.92 ± 4.62(353)	0.008	0.001
	AT	7.58 ± 4.81(154)		
	TT	11.80 ± 8.85(14)		
	AA	6.92 ± 4.62(353)	0.023	0.001
	AT + TT	7.93 ± 5.35(168)		
rs12444338	GG	8.64 ± 5.45(230)	1.43 × 10^−10^	3 × 10^−8^
	GT	6.30 ± 3.93(228)		
	TT	5.38 ± 4.50(62)		
	GG	8.64 ± 5.45(230)	3.09 × 10^−10^	2.23 × 10^−11^
	GT + TT	6.11 ± 4.07(290)		
rs4783244	GG	8.62 ± 5.42(234)	3.36 × 10^−10^	4.08 × 10^−8^
	GT	6.31 ± 3.95(226)		
	TT	5.45 ± 4.48(62)		
	GG	8.62 ± 5.42(234)	4.62 × 10^−10^	1.95 × 10^−11^
	GT + TT	6.12 ± 4.08(288)		
rs12051272	GG	8.58 ± 5.44(235)	2.78 × 10^−10^	7.45 × 10^−9^
	GT	6.40 ± 3.98(229)		
	TT	5.15 ± 4.38(57)		
	GG	8.58 ± 5.44(235)	2.25 × 10^−9^	1.57 × 10^−10^
	GT + TT	6.15 ± 4.08(286)		

N: number of subjects; P value, Unadjusted;

P*°*value, adjusted for age, sex, body mass index, and current smoking status.

A dominant inheritance model revealed a significant association between the minor alleles of rs12444338, rs4783244, and rs12051272 and lower adiponectin levels (*P* = 2.23 × 10^−11^, *P* = 1.95 × 10^−11^, and *P* = 1.57 × 10^−10^, respectively) and between the minor alleles of rs11646213 and higher adiponectin levels (*P* = 0.001). In addition, haplotype analysis revealed an association among the three haplotypes AGGG, ATTT, and TGGG, accounting for 97.73% of all of the inferred haplotypes, and the adiponectin levels (*P* = 1.25 × 10^−4^, *P* = 3.78 × 10^−13^ and *P* = 7.67 × 10^−5^, respectively) (Table C in S1 File). Multivariate linear regression analysis revealed the two promoter gene variants rs12444338 and rs11646213 to be independent predictors of the adiponectin levels (*P* = 2.22 × 10^−7^ and *P* = 0.01, respectively) (Table D in S1 File).

### Associations between adiponectin levels or the CDH13 gene variant rs12051272 and metabolic syndrome-related phenotypes

Table 3 presents the associations of the adiponectin levels and the *CDH13* gene variant rs12051272 with metabolic syndrome and its related phenotypes.

**Table 3:** Association of *CDH13* rs12051272 genotypes, serum adiponectin levels and metabolic syndrome-related phenotypes.

	*CDH13* genotypes*		serum adiponectin		*CDH13* genotypes, serum adiponectin adjusted		serum adiponectin, *CDH13* genotypes adjusted	
	B (95% CI)	P value	B (95% CI)	P value	B (95% CI)	P value	B (95% CI)	P value
	Adjustments for age and sex							
Systolic BP	−0.035(−2.615–2.544)	0.978	−7.032(−11.907 to—2.157)	0.005	−1.175(−3.862–1.512)	0.391	−7.297(−12.468 to −2.125)	0.006
Diastolic BP	−0.776(−2.446–0.895)	0.362	−5.456(−8.627 to −2.285)	0.001	−1.718(−3.450–0.014)	0.052	−6.037(−9.370 to −2.704)	4.07 × 10^−4^
Fasting plasma glucose	−1.315(−5.172–2.542)	0.503	−15.190(−22.370 to −8.011)	3.77 × 10^−5^	−4.093(−8.062 to −0.125)	0.043	−17.794(−25.431 to −10.156)	5.91 × 10^−6^
Fasting serum insulin	0.359(−0.493–1.212)	0.408	−4.907(−6.470 to −3.344)	1.38 × 10^−9^	−0.445(−1.310–0.419)	0.312	−5.152(−6.815 to −3.488)	2.28 × 10^−9^
HOMA-IR index	0.062(−0.174–0.297)	0.607	−1.507(−1.935 to −1.080)	1.26 × 10^−11^	−0.191(−0.428–0.045)	0.112	−1.621(−2.076 to −1.167)	7.59 × 10^−12^
QUICKI	−0.002(−0.006–0.003)	0.462	0.034(0.026–0.041)	6.23 × 10^−17^	0.004(−0.0002–0.008)	0.064	0.036(0.028–0.044)	5.51 × 10^−17^
HDL-cholesterol	−0.003(−0.021–0.014)	0.705	0.172(0.142–0.202)	1.87 × 10^−26^	0.026(0.009–0.042)	0.002	0.186(0.155–0.218)	1.04 × 10^−27^
Triglyceride	−0.010(−0.052–0.032)	0.634	−0.323(−0.397 to −0.249)	1 × 10^−16^	−0.068(−0.108 to −0.027)	0.001	−0.368(−0.446 to −0.290)	4.38 × 10^−19^
Metabolic syndrome**	0.879(0.538–1.437)	0.607	0.028(0.010–0.081)	3.74 × 10^−11^	0.443(0.251–0.783)	0.005	0.016(0.005–0.053)	4.15 × 10^−12^
	Adjustments for age, sex, BMI, and current smoking status							
Systolic BP	−1.283(−3.722–1.155)	0.302	−0.741(−5.619–4.137)	0.766	−1.430(−3.970–1.109)	0.269	−1.071(−6.194–4.051)	0.681
Diastolic BP	−1.597(−3.159–0.035)	0.045	−1.352(−4.511–1.807)	0.401	−1.861(−3.487 to −0.236)	0.025	−1.929(−5.207–1.349)	0.248
Fasting plasma glucose	−1.889(−5.768–1.990)	0.339	−14.146(−21.748 to −6.544)	2.82 × 10^−4^	−4.183(−8.159 to −0.207)	0.039	−16.730(−24.750 to −8.710)	4.84 × 10^−5^
Fasting serum insulin	−0.175(−0.954–0.603)	0.658	−2.521(−4.052 to −0.990)	0.001	−0.563(−1.364–0.239)	0.168	−2.827(−4.444 to −1.210)	0.001
HOMA-IR index	−0.075(−0.293–0.142)	0.496	−0.921(−1.346 to −0.496)	2.47 × 10^−5^	−0.219(−0.441–0.003)	0.054	−1.046(−1.494 to −0.598)	5.73 × 10^−6^
QUICKI	0.001(−0.002–0.005)	0.487	0.021(0.014–0.029)	2.6 × 10^−8^	0.005(0.001–0.008)	0.019	0.024(0.016–0.032)	3.81 × 10^−9^
HDL-cholesterol	0.005(−0.012–0.022)	0.571	0.149(0.117–0.180)	1.87 × 10^−19^	0.027(0.011–0.044)	0.001	0.164(0.132–0.197)	2.58 × 10^−21^
Triglyceride	−0.030(−0.070–0.009)	0.134	−0.266(−0.341 to −0.190)	1.38 × 10^−11^	−0.073(−0.112 to −0.034)	2.39 × 10^−4^	−0.314(−0.393 to −0.236)	2.24 × 10^−14^
Metabolic syndrome**	0.61 (0.36–1.05)	0.074	0.042(0.013–0.129)	3.74 × 10^−8^	0.32 (0.17–0.60)	3.34 × 10^−4^	0.019(0.005–0.069)	9.85 × 10^−10^

Abbreviations as in Table 1

*CDH13 genotypes: dominant inheritance model was used (*GG vs*. *GT + TT* for rs12051272).

** For metabolic syndrome, the lanes denote the odds ratios (95% confidence interval [CI]).

The rs12051272 SNP was used for analysis because of the nearly complete linkage between the two studied intron 1 polymorphisms. After adjustment for age and sex, significant correlations were observed between the adiponectin levels and all of the metabolic syndrome-related phenotypes (all *P* < 0.01) (Table 3). After further adjustment for body mass index (BMI) and current smoking status, significant associations with adiponectin levels remained in most metabolic phenotypes, except the BP status.

After adjustments for age and sex or further adjustments for BMI and current smoking status, no significant correlations were observed between rs12051272 and the related metabolic phenotypes, whereas a borderline significant association was observed between rs12051272 and diastolic BP (Table 3). After further adjustment for adiponectin levels, significant associations were observed between a minor allele of rs12051272 with a more favorable metabolic profile, including higher insulin sensitivity and HDL cholesterol levels and lower diastolic BP and circulating levels of fasting plasma glucose, and triglycerides (*P* = 0.019, 0.001, 0.025, 0.039 and 2.39 × 10^−4^, respectively).

The serum adiponectin levels were significantly lower in participants with diabetes mellitus, metabolic syndrome, and insulin resistance (Table 3 and Table E in S1 File). For the analysis between the rs12051272 genotypes in the dominant model and metabolic syndrome, a significant association was evident only after further adjustment for adiponectin levels (*P* = 3.34 × 10^−4^, odds ratio = 0.32, and 95% confidence interval = 0.17–0.60) (Table 3). However, no significant association was observed between the rs12051272 genotypes and the other risk factors (Table E in S1 File).

### Adiponectin levels suppress the associations of CDH13 genotypes and metabolic profiles with biomarker levels and metabolic syndrome

Four criteria were used for establishing the mediation and suppression effects (Table 4).

**Table 4:** Mediation tests for adiponectin levels on the association between *CDH13* genotypes and metabolic phenotypes.

		Diastolic BP	Glucose	QUICKI	HDL-cholesterol	Triglyceride	Metabolic syndrome
**Criterion 1**	α						
	regression coefficient	−0.137	−0.137	−0.137	−0.137	−0.137	−0.137
	Standard error	0.021	0.021	0.021	0.021	0.021	0.021
	*P* value	1.57 × 10^−10^	1.57 × 10^−10^	1.57 × 10^−10^	1.57 × 10^−10^	1.57 × 10^−10^	1.57 × 10^−10^
**Criterion 2**	β						
	regression coefficient	1.929	−0.056	0.024	0.164	−0.314	−3.946
	Standard error	1.669	0.012	0.004	0.017	0.040	0.646
	*P* value	0.248	4.84 × 10^−5^	3.81 × 10^−9^	2.58 × 10^−21^	2.24 × 10^−14^	9.85 × 10^−10^
	γ’						
	regression coefficient	−1.861	−0.013	0.005	0.027	−0.073	−1.136
	Standard error	0.827	0.006	0.002	0.008	0.020	0.317
	*P* value	0.025	0.039	0.019	0.001	2.39 × 10^−4^	3.34 × 10^−4^
**Criterion 3**	αβ+γ’						
	regression coefficient	−1.597	−0.005	0.001	0.005	−0.030	−0.492
	Standard error	0.795	0.006	0.002	0.009	0.020	0.275
	*P* value	0.045	0.339	0.487	0.571	0.134	0.074
**Criterion 4**	αβ						
	regression coefficient	0.264	0.008	−0.003	−0.022	0.043	0.541
	Standard error	0.232	0.002	0.0007	0.004	0.009	0.121
	*P* value (sobel test)	0.255	5.2 × 10^−4^	1 × 10^−5^	7 × 10^−8^	5.2 × 10^−7^	8.24 × 10^−6^

*CDH13* genotypes represent rs12051272, which was analyzed in the dominant models, α: unstandardized coefficient for the association between the *CDH13* genetic variants and log adiponectin levels; β: unstandardized coefficient for the association between adiponectin and metabolic phenotypes, such as diastolic BP, glucose levels, QUICKI, HDL cholesterol, triglyceride levels and metabolic syndrome (when adjusted for the *CDH13* genetic variants). Direct effect = γ’, Total effect = αβ + γ’, Mediation (indirect) effect = αβ

**Fig 1:**
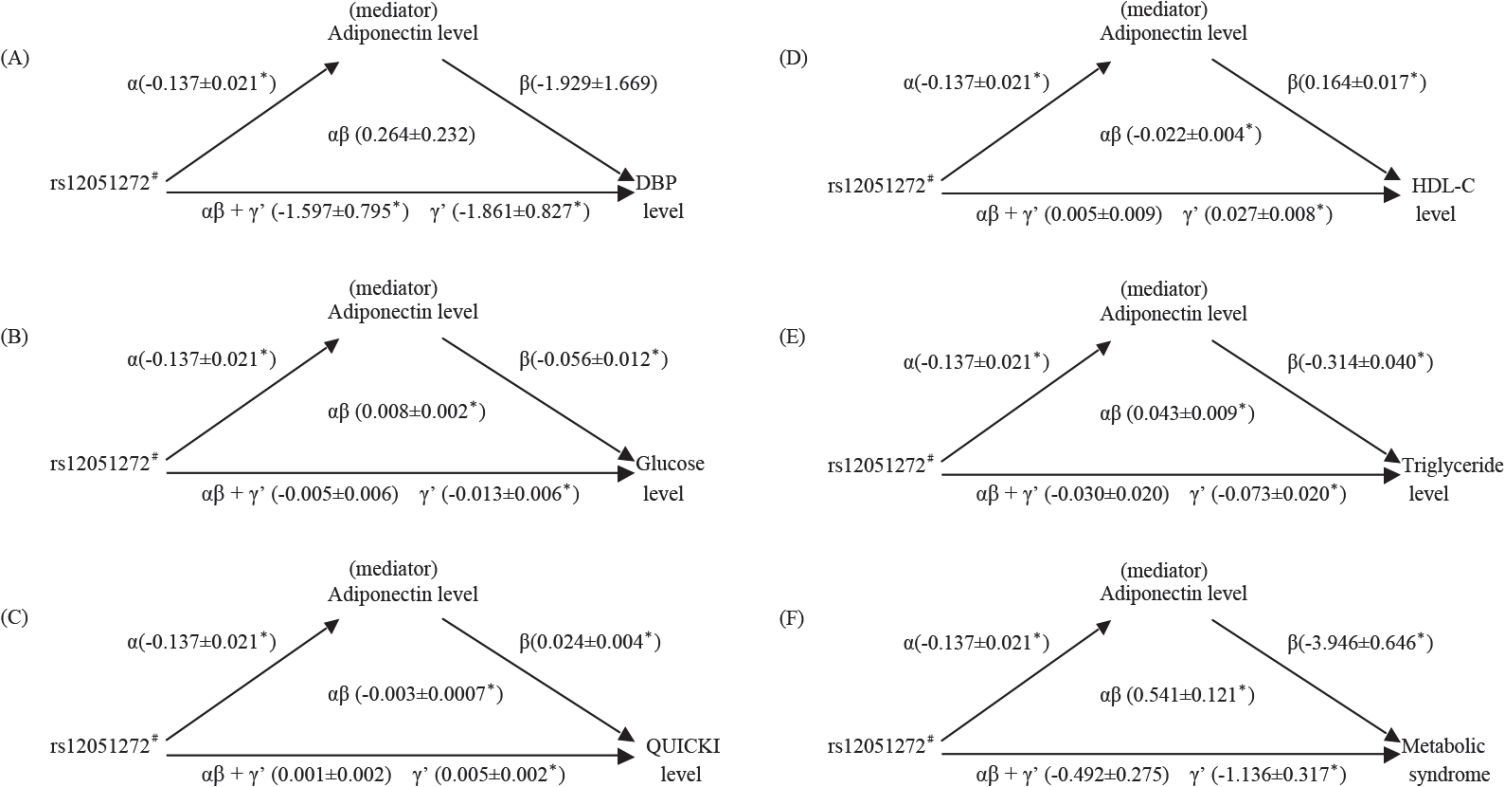
A three-variable mediation model. Adiponectin levels as a mediator of the association between the *CDH13* genotype and the metabolic syndrome and metabolic syndrome-related phenotypes. Linear or binary regression models were used to assess the following path associations: in Fig 1 (F): (α) relationship between the *CDH13* genotype and adiponectin, (β) relationship between adiponectin and metabolic syndrome, (αβ + γ’) relationship between the *CDH13* genotype and metabolic syndrome, and (γ’) relationship between the *CDH13* genotype and metabolic syndrome after adjustment for adiponectin levels. Each estimate along the path represents the unstandardized β coefficient from the regression model. The results indicate that after adjustment for adiponectin levels, the CDH13 genotype exhibited a stronger association with the metabolic syndrome. The direct effects (γ’) of the *CDH13* genotype on the metabolic syndrome (−1.136) were greater than the total effects (αβ + γ’) (−0.492) and revealed similar magnitudes and opposite signs than those of the mediation effects (αβ) (0.541), suggesting significant mediation (suppression) by the adiponectin levels. All of the models were adjusted for age, sex, BMI, and current smoking status. °P < 0.05. In addition, the other metabolic syndrome-related phenotypes exhibited similar suppression effects (B, C, D, and E). ^#^ *CDH13* genotypes: dominant inheritance model was used (*GG vs*. *GT + TT* for rs12051272).

BP, blood pressure; HDL, high-density lipoprotein; QUICKI, quantitative insulin sensitivity check index;

Only diastolic BP did not match the criteria for the suppression effect conferred by the adiponectin levels. In brief, the *CDH13* genotypes exhibited significant associations with the adiponectin levels (criterion 1), which in turn, conferred considerably positive effects on the fasting plasma glucose levels, QUICKI, HDL cholesterol and triglyceride levels and metabolic syndrome (criterion 2). The total effects of the *CDH13 g*enotypes on each metabolic phenotype were −0.005, 0.001, 0.005, −0.030, and −0.492, respectively, with all nonsignificant P values (criterion 3). The Sobel tests for mediation on the results of each metabolic phenotype revealed the following: z = 3.47, 4.7, 5.62, 4.78 and 4.47 (*P* = 5.2 × 10^−4^, 1.0 × 10^−5^, 7.0 × 10^−8^, 5.2 × 10^−7^, and 8.24× 10^−6^, respectively) (criterion 4). Moreover, the direct effects (γ’) of rs12051272 on each metabolic phenotype, except diastolic BP, were greater than their total effects (αβ + γ’) and revealed similar magnitudes and opposite signs than those of the mediation effects (αβ), thereby demonstrating a suppression effect in this model (Fig 1).

## Discussion

The present study data indicated a significant association between *CDH13* genotypes/haplotypes and adiponectin levels. In addition, the *CDH13* genotypes exhibited a significant association with insulin resistance, metabolic syndrome and related metabolic phenotypes. As observed in the mediation analysis, adiponectin levels suppress the association between the *CDH13* genotypes and the various metabolic phenotypes and metabolic syndrome. Overall, a complex relationship was observed among the *CDH13* locus variants and metabolic syndrome, and these results suggested that the *CDH13* gene variants play a crucial role in the genetic determinants of metabolic syndrome and related metabolic phenotypes. In addition, this investigation further supports the speculation that the suppression effect may play a crucial role in biological science.

### Evidence suggesting association of adiponectin levels and adiponectin-related gene variants with metabolic phenotypes and metabolic syndrome

The metabolic syndrome is a complex syndrome with clustering of multiple cardiovascular risk factors. Central obesity has been suggested to be the cardinal feature of the metabolic syndrome, and a newer model for the pathogenesis of metabolic syndrome has revealed that this syndrome is associated with dysregulated adipose tissues and inflammatory cytokine overexpression [31, 32]. Several studies, including the present study, have indicated a negative correlation between plasma adiponectin levels and insulin resistance measures in addition to the risk of metabolic syndrome [5, 19]. Recent genome-wide association studies evaluating the genetic determinants of adiponectin levels, including *ADIPOQ*, *CDH13* and *WDR11-FGFR2* gene variants, have reported considerable associations with various metabolic phenotypes, including insulin resistance, diabetes mellitus, metabolic syndrome, and cardiovascular disease [10, 14–16, 19]. These results emphasize the importance of adiponectin-related genes as genetic risk factors for metabolic abnormalities and atherosclerotic cardiovascular disorders.

### Evidence and possible mechanisms of the associations between CDH13 genotypes/haplotypes and adiponectin levels

A recent meta-analysis revealed the *CDH13* gene region to be the most crucial locus associated with adiponectin levels [16]. In addition, the *CDH*13 rs4783244 polymorphism has been demonstrated to be the strongest statistically associated SNP with adiponectin levels in several investigations conducted in East Asia [14, 15, 19]. The present data revealed nearly complete linkage between the *CDH13* promoter region SNP rs12444338 polymorphism and the two studied *CDH13* intron 1 polymorphisms. The above 3 studied polymorphisms revealed decrease adiponectin levels in the participants with minor alleles of the polymorphisms. Morisaki et al. suggested that the phenotype-affecting haplotype tagged by SNP rs12051272 may affect the T-cadherin baseline levels in the tissues that capture the free adiponectin molecules in the plasma, thereby reducing the plasma HMW adiponectin levels [19]; this is consistent with other studies, which have demonstrated that the ablation of the T-cadherin receptor increases the plasma adiponectin levels in mice [17, 18]. However, based on a reporter assay, Jee et al. reported that the major allele of the promoter SNP rs12444338 leads to a 2.2-fold increase in the *CDH13* expression [13]; this allele also increased the adiponectin levels in the present investigation. These contradictory results raise questions regarding the role of changes in *CDH13* gene expression with *CDH13* genotypes as the major mechanism affecting the adiponectin levels. Another possibility is that the effects of *CDH13* genotypes may be related to the increased binding of the HMW adiponectin to *CDH13* receptors, which in turn, reduced the circulating adiponectin levels, as hypothesized by Gao et al. [15]. Alternatively, a possibility can not be excluded that the association of the studied *CDH13* SNPs with adiponectin levels may be due to the linkage disequilibrium with other polymorphisms or mutations that are responsible for these associations.

### Mediation analysis and suppression effects

Suppression effects have been seldom reported in biological science. In mediation hypotheses, a suppression effect may be suggested if the statistical removal of a mediational effect could increase the magnitude of the relationship between the independent and the dependent variables. A recent study revealed that CRP mediates the effects of apolipoprotein E on cytomegalovirus infection, demonstrating that CRP is a key suppressor of the relationship between the ε4 and the cytomegalovirus antibody levels [33]. Our previous study revealed that sE-selectin levels exhibit a suppression effect on the association between the ABO blood-group genotypes and the triglyceride to HDL cholesterol ratio [25], which strengthens the importance of the relationship between ABO and atherogenesis. The present study indicated that the adiponectin levels act as a suppressor of the association between *CDH13* variants and various metabolic phenotypes. Overall, these findings indicate the crucial role of the mediation effect in biological science, and additional mediation analysis may elucidate further genetic associations in the biomarker levels or disease relationship.

### Mechanisms of association of CDH13 genotypes/haplotypes with metabolic phenotypes and metabolic syndrome

The data from several previous studies and our research have consistently demonstrated the association of a minor allele of *CDH13* gene variants with lower adiponectin levels and, contrarily, with a more favorable metabolic profile. Previous studies have suggested that almost all of the metabolic effects of adiponectin are conferred by adiponectin R1/R2 receptor [34]. By contrast, the binding of adiponectin to T-cadherin can activate the nuclear factor-kB signaling pathway, which plays a key role in inflammation and serves as a link between obesity and vascular disease [35]. T-cadherin not only competes with the adiponectin R1/R2 receptors for adiponectin binding but also interferes with the coupling of both receptors to their downstream intracellular targets [36]. Thus, a minor allele of the intron 1 polymorphisms, through close linkage to promoter polymorphisms, may decrease the *CDH13* gene and T-cadherin expressions, which result in decreased competition with other adiponectin receptors and increase the coupling of adiponectin R1/R2 receptors with downstream intracellular targets as well as the metabolic effects of adiponectin. As proposed previously [15], chronic low levels of adiponectin may be associated with increased adiponectin sensitivity and adiponectin R1/R2 expression, which also increase the metabolic effects of adiponectin. Consistent with the proposed mechanisms, a previous study reported that chronic elevation of plasma adiponectin levels downregulated the AdipoR2 expression in murine adipose tissues [37]. Reportedly, the adiponectin R1/R2 expression was upregulated in insulin-resistant women with polycystic ovary syndrome [38], who were expected to exhibit low circulating adiponectin levels. This hypothesis can be supported by the mediation analysis performed in this study, which indicates that adiponectin acts as a suppressor of the association between the *CDH13* genotypes/haplotypes and the various metabolic phenotypes and metabolic syndrome.

### Limitations

Only four *CDH13* SNPs were analyzed in this investigation, and the incomplete genotyping may not be representative of all *CDH13* gene haplotypes. A stringent application of Bonferroni correction for multiple tests marginalized the statistical significance of a part of our results. Therefore, cautious and conservative data interpretation is warranted. However, the consistent association of favorable metabolic phenotypes and a lower risk of metabolic syndrome in the allele with lower adiponectin levels, which is compatible with previous reports, suggested that the association may not be due to chance. In addition, our study was obtained from only one sample population. The biology of the mediation effect remains somewhat speculative since supportive experiment is difficult. Therefore, replication in a second cohort with larger sample size and Mendelian randomization design would improve strength of the analysis.

## Conclusion

In conclusion, our data reveal a significant association of *CDH13* locus variants with not only adiponectin levels but also metabolic phenotypes and metabolic syndrome. Furthermore, adiponectin levels acts as a suppressor on the association between *CDH13* variants and various metabolic phenotypes. These results suggest that the suppression effect may be crucial in biological science and provide further evidence of the association between *CDH13* and the risks of metabolic syndrome and atherosclerotic cardiovascular disease.

## Supporting Information

S1 File Table A, Primer sequences. Table B, Linkage disequilibrium between CDH13 genetic polymorphisms. Table C, *CDH13* haplotypes and adiponectin levels. Table D, Adiponectin levels: stepwise linear regression analysis, including genotypes. Table E, Association of the *CDH13* gene variant rs12051272 and circulating adiponectin levels with cardiovascular risk factors.
(DOC)
