# Supplementary material for: Association of CDH13 Genotypes/Haplotypes with Circulating Adiponectin Levels, Metabolic Syndrome, and Related Metabolic Phenotypes: The Role of the Suppression Effect
Source: PLoS One. 2015 Apr 13;10(4):e0122664. doi: 10.1371/journal.pone.0122664 (PMC4395292; doi:10.1371/journal.pone.0122664)
Supplement: S1 File — Table B, Linkage disequilibrium between CDH13 genetic polymorphisms. Table C, CDH13 haplotypes and adiponectin levels. Table D, Adiponectin levels: stepwise linear regression analysis, including genotypes. Table E, Association of the CDH13 gene variant rs12051272 and circulating adiponectin levels with cardiovascular risk factors. (DOC) [file pone.0122664.s001.doc]

Supplementary Table A. Primer sequences

| Gene | SNP number | Position | Location | Minor allele | MAF | HWE | Primer sequence |
| --- | --- | --- | --- | --- | --- | --- | --- |
| CDH13 | rs11646213 | 82609046 |  | T | 0.173 | 0.825 | TaqMan SNP Genotyping Assays |
| CDH13 | rs12444338 | 82626550 | nearGene-5 | T | 0.337 | 0.968 | TaqMan SNP Genotyping Assays |
| CDH13 | rs4783244 | 82628663 | Intron | T | 0.336 | 0.955 | TaqMan SNP Genotyping Assays |
| CDH13 | rs12051272 | 82629683 | Intron | T | 0.329 | 1 | TaqMan SNP Genotyping Assays |

MAF: minor allele frequency; HWE: Hardy–Weinberg equilibrium

Supplementary Table B. Linkage disequilibrium between CDH13 genetic polymorphisms

|  | rs11646213 | rs12444338 | rs4783244 | rs12051272 |
| --- | --- | --- | --- | --- |
| rs11646213 | - | 0.8881 | 0.8913 | 0.8874 |
| rs12444338 | - | - | 0.9826 | 0.9955 |
| rs4783244 | - | - | - | 0.9867 |
| rs12051272 | - | - | - | - |

The values represent D’

Supplementary Table C. *CDH13* haplotypes and adiponectin levels

|  |  |  | Adiponectin levels | |
| --- | --- | --- | --- | --- |
|  | Haplotype | Frequency | Coefficient | P value |
| H1 | AGGG | 49.12% | 0.1151 | 1.25 × 10^−4^ |
| H2 | ATTT | 31.80% | −0.2379 | 3.78 × 10^−13^ |
| H3 | TGGG | 16.81% | 0.1652 | 7.67 × 10^−5^ |

SNP1: rs11646213, SNP2: rs12444338, SNP3: rs4783244, and SNP4: rs12051272. Coefficients and P values were estimated based on the haplotype trend regression analysis implemented in the HelixTree program. The examined haplotype was compared with all of the unexamined haplotypes; P values were adjusted for age, sex, body mass index, and current smoking status.

Supplementary Table D. Adiponectin levels: stepwise linear regression analysis, including genotypes.

| Variable | Beta | R^2a^ | P value |
| --- | --- | --- | --- |
| Sex | 0.192 | 0.151 | 5.87 × 10^−18^ |
| BMI | −0.022 | 0.234 | 1.11 × 10^−12^ |
| *CDH13* rs12444338 | −0.016 | 0.277 | 2.22 × 10^−7^ |
| age | 0.004 | 0.297 | 2.8 × 10^−4^ |
| *CDH13* rs11646213 | 0.015 | 0.299 | 0.010 |

Supplementary Table E. Association of the *CDH13* gene variant rs12051272 and circulating adiponectin levels with cardiovascular risk factors

|  |  | *Adiponectin levels* | | rs12051272 (GG *vs*. GT + TT) | | | |
| --- | --- | --- | --- | --- | --- | --- | --- |
|  |  | Mean ± SD (N) | P1 value | Odds ratio (95% CI) | P1 value | Odds ratio (95% CI) | P2 value |
| Current smoking status* | Noncurrent  Current | 7.7 ± 5.03 (426)  5.43 ± 3.82 (104) | 0.332 | 1.27 (0.78–2.06) | 0.332 | 1.20 (0.73–1.99) | 0.468 |
| Hypertension | Without  With | 7.3 ± 4.96 (479)  6.82 ± 4.24 (51) | 0.865 | 0.60 (0.31–1.14) | 0.118 | 0.59 (0.30–1.16) | 0.127 |
| Diabetes mellitus | Nil  Yes | 7.33 ± 4.89 (517)  4.46 ± 4.59 (13) | 0.001 | 0.62 (0.20–1.91) | 0.403 | 0.36 (0.11–1.20) | 0.096 |
| Metabolic syndrome** | Nil  Yes | 7.71 ± 5.02 (454)  4.55 ± 2.89 (76) | 2.08 × 10^−8^ | 0.61 (0.36–1.05) | 0.074 | 0.32 (0.17–0.60) | 3.34× 10^−4^ |
| Insulin resistance | Nil  Yes | 7.94 ± 5.06 (411)  4.89 ± 3.33 (119) | 1.61 × 10^−7^ | 1.13 (0.71–1.79) | 0.601 | 0.75 (0.45–1.23) | 0.254 |

P1 value, adjusted for age, sex, body mass index, and current smoking status

P2 value, adjusted for age, sex, body mass index, current smoking status, and adiponectin levels

* P1 value, adjusted for age, sex and body mass index

** The P2 values for the dominant model of rs12444338, rs4783244, and rs11646213 were 0011, 0.001, and 0.031, respectively.
